# Fat embolism after intraosseous catheters in pediatric forensic autopsies

**DOI:** 10.1007/s00414-022-02848-4

**Published:** 2022-06-30

**Authors:** C. Castiglioni, A. Carminati, T. Fracasso

**Affiliations:** 1 University Center of Legal Medicine, chemin de la Vulliette n. 4, 1000 Lausanne, Switzerland; 2 University Center of Legal Medicine, rue Michel-Servet n. 1, 1206, Geneva, Switzerland

**Keywords:** Pediatric resuscitation, Intraosseous catheters, Autopsy, Pulmonary fat embolism

## Abstract

In our center, we performed the autopsy of a child who died from drowning and presented, at autopsy, a major pulmonary fat embolism (PFE). A cardiopulmonary resuscitation (CPR) was performed, including infusion by intraosseous catheter (IIC). No other traumatic lesions and diseases classically related to a risk of PFE were detected. According to some animal studies, we considered the IIC as the only possible cause for PFE. However, we could not find literature to confirm this hypothesis in humans, especially in a pediatric population. To verify the occurrence of PFE after IIC in a pediatric population, we retrospectively selected 20 cases of pediatric deaths autopsied in our center, in which a CPR was performed, without bone fractures or other possible causes of PFE: 13 cases with IIC (group A) and 7 cases without IIC (group B). Several exclusion criteria were considered. The histology slides of the pulmonary tissue were stained by Oil Red O. PFE was classified according to the Falzi scoring system. In group A, 8 cases showed PFE: 4 cases with a score 1 of Falzi and 4 cases with a score 2 of Falzi. In group B, no case showed PFE. The difference between the two groups was statistically significant. The results of our study seem to confirm that IIC can lead to PFE in a pediatric population and show that the PFE after IIC can be important (up to score 2 of Falzi). To the best of our knowledge, our study is the first specifically focused on the occurrence of PFE after IIC in a pediatric population by using autoptic data.

## Introduction

Fat embolism is a well-known phenomenon in orthopedic surgery and in forensic medicine, essentially in cases of blunt trauma with bone fractures, involving mainly the lungs and more rarely the brain or the kidneys. In forensic medicine, in some cases of traumatic deaths, the occurrence of pulmonary fat embolism (PFE) can be considered a vital reaction [1]. Some studies showed that PFE can also be found without bone fractures, in cases with corticosteroid treatment, fatty liver, diabetes, osteomyelitis, burns, liposuction, cardiopulmonary bypass surgery, decompression sickness, parenteral lipid infusion, hemorrhagic pancreatitis, carbon tetrachloride poisoning, massive hepatic necrosis with fatty liver, heat exposure, and sickle-cell disease or in cases of diffuse soft tissue contusions [2–5]. In the end, it is known that PFE can be detected after cardio-pulmonary resuscitation (CPR) by external cardiac massage with rib cage fractures [6, 7].

In our center, we performed the autopsy of a child who died from drowning and presented, at histological post-mortem examination of lungs, a major pulmonary fat embolism, with a score 2 according to Falzi scoring system [8]. The child was resuscitated for approximately 1 h by external cardiac massage, airway intubation, and infusion by two intraosseous catheters in the tibial region, without return to a spontaneous circulation. No traumatic lesions and no natural diseases were observed after multiple post-mortem investigations (PMCT, MRI, autopsy, histological examination, and biochemical analysis). In the absence of traumatic lesions or preexisting pathologies classically related to a risk of PFE, we considered the infusion by intraosseous catheter (IIC) as the only possible explanation for PFE. The PFE after IIC is described in some animal models [9–11], but is only poorly described in humans, especially in a pediatric population.

For this reason, we have carried out the present study to verify the occurrence of PFE after IIC in a pediatric resuscitated population. The pediatric population is an ideal population for this type of study, as thoracic fractures after CPR are less frequent than in adults, thus limiting this potential confounding factor.

## Materials and methods

Pediatric forensic autopsy cases of the University Center of Legal Medicine of Lausanne and Geneva (Switzerland) were reviewed from 2014 to 2020. We selected pediatric cases of deaths (age < 15 years) in which a CPR was performed. The cases were divided in two groups, group A with IIC and group B without IIC. In all cases, an autopsy had been performed, preceded by a PMCT and followed by toxicological, biochemical, and histological analysis.

To avoid any alternative cause of PFE, we excluded cases with bones fractures, large soft tissue contusions, preexisting disease correlated to a risk of PFE (for example fatty liver, diabetes, sickle-cell disease), and corticosteroid treatment. As exclusion criteria, we also used a post-mortem interval > 72 h and a survival time interval > 72 h.

For each case, we recovered samples of the five lung lobes that had been taken during the autopsy and stored in formaldehyde in our archives. To perform a histological examination specifically aimed at the search for PFE, the frozen histological slides were stained by a specific staining for fat droplets, the Oil Red O staining (ORO).

The ORO-stained slides were examined by two examiners, with a final consensual evaluation. To quantify the degree of PFE, a score was assigned to each case by using the Falzi scoring system, adapted by Janssen (Table 1) [8, 12].

**Table 1:** Falzi scoring system for PFE, adapted by Janssen

Score	Emboli present
0—no FE	Not fat embolism (punctiform when present)—sporadic
1—mild FE	Light fat embolism drop-shaped, sporadic in every microscopic field
2—distinct FE	Multiple, disseminated, sausage shaped or rounded
3—massive FE	Every microscopic field, antler shaped and numerous everywhere

The results of the two groups were compared to search for a statistically significant difference by using a Student *t*-test.

## Results

We selected a total of 20 cases of pediatric deaths in which a CPR was performed: 13 cases with IIC (group A) and 7 cases without IIC (group B). In group A, 7 cases received one tibial intraosseous catheter and 6 cases two tibial intraosseous catheters.

The median age was 2.3 years for the group A and 7.3 years for the group B. The causes of deaths in group A were drowning (*n* = 4), hanging (*n* = 1), sudden infant death (*n* = 4), infection (*n* = 2), food inhalation (*n* = 1), and febrile seizures (*n* = 1). The causes of deaths in group B were drowning (*n* = 2), hanging (*n* = 2), sudden infant death (*n* = 2), and infection (*n* = 1). The demographic data and causes of death are reported in Table 2.

**Table 2:** Demographic data and causes of death

	*n*	Mean age	Sex	Cause of death
Group A	13	2.3 years (range 1 month–14 years)	7 ♂ 6 ♀	4 drowning 1 hanging 4 sudden infant death 2 infection 1 food bronchoaspiration 1 febrile seizures
Group B	7	7.3 years (range 1 month–15 years)	4 ♂ 3 ♀	2 drowning 2 hanging 2 sudden infant death 1 infection

**Fig. 1:**
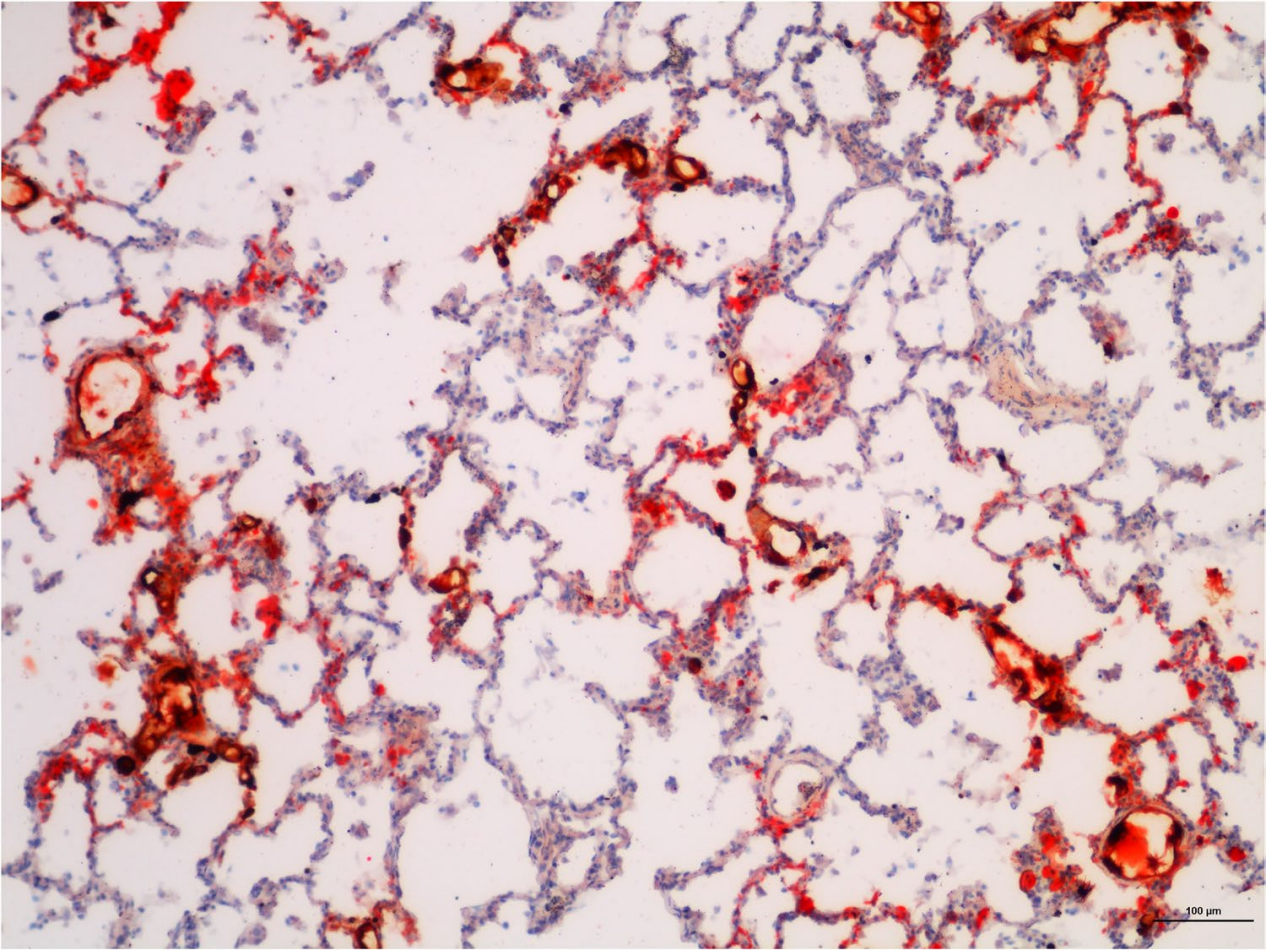
Frozen pulmonary tissue stained Oil Red O (10 ×): important PFE (red spots) in a case of group A

In group A, 8 cases showed PFE (61%), 4 with a score 1 of Falzi and 4 with a score 2 of Falzi (Fig. 1). In group B, no cases showed PFE. The difference between the two groups was statistically significant (*p* = 0.0119). The results are shown in Table 3 for group A and Table 4 for group B.

**Table 3:** Results for the group A

Group A	Age	Sex	Cause of death	Number of IC	Grade of PFE (score of Falzi)
*Case n° 1*	2 years	♀	Infection	2	2
*Case n° 2*	2 years	♀	Drowning	2	2
*Case n° 3*	3 years	♀	Febrile seizures	1	2
*Case n° 4*	14 years	♀	Hanging	1	2
*Case n° 5*	1 year	♂	Food bronchoaspiration	2	1
*Case n° 6*	2 years	♂	Drowning	2	1
*Case n° 7*	2 years	♂	Drowning	1	1
*Case n° 8*	4 months	♂	Sudden infant death	1	1
*Case n° 9*	8 months	♀	Sudden infant death	2	0
*Case n° 10*	1 month	♀	Sudden infant death	2	0
*Case n° 11*	4 months	♂	Sudden infant death	1	0
*Case n° 12*	1 years	♂	Infection	1	0
*Case n° 13*	2 years	♂	Drowning	1	0

**Table 4:** Results for the group B

Group B	Age	Sex	Cause of death	Falzi scoring system
*Case n° 1*	15 years	♂	Hanging	0
*Case n° 2*	15 years	♂	Drowning	0
*Case n° 3*	2 months	♀	Sudden infant death	0
*Case n° 4*	1 month	♀	Sudden infant death	0
*Case n° 5*	4 years	♂	Drowning	0
*Case n° 6*	14 years	♀	Hanging	0
*Case n° 7*	3 years	♂	Infection	0

In group A, we did not find any correlation between PFE and other factors such as the age or the number of intraosseous catheters.

## Discussion

The use of intraosseous devices in critical patients is known since 1922 and is currently the standard alternative to intravenous access, especially in the pediatric population. Many techniques can be used for intraosseous infusions, the three main types being the manual, the impact-driven, and the power-driven needles [13, 14]. The needle passes through the bone cortex into the marrow cavity, where the infusion, usually of an electrolytic solution, takes place. Due to a smaller medullary cavity in young patients, intraosseous catheter placement is more difficult than in adults, and complications are more frequent. The main complications that have been described in the literature are extravasation of fluid, necrosis, limb ischemia, infection, fracture, or compartment syndrome [13, 15, 16].

In the literature, PFE following IIC has been investigated in some studies on animal models (dogs, piglets, and swines), sometimes with contradictory results. In two studies on piglets and swines, the authors did not find PFE after IIC [17, 18]. On the contrary, in another study on piglets, the authors found the presence of PFE after IIC in about 30% of the cases [9]. Furthermore, in this study, the authors tested different methods of IIC and concluded that the volume and pressure of infusion do not influence the incidence of PFE. In another study on swines, the authors concluded that PFE is a common consequence of IIC and that its magnitude is influenced by the site of cannulation and the infusion forces [10].

Concerning humans, in a study on an animal model (dogs), the authors mentioned two cases of children who had received IIC and in which they have found PFE, but the link between PFE and IIC in these cases was not further discussed [11]. To the best of our knowledge, this phenomenon has never been well investigated until now. In a 2010 review about intraosseous infusions for pediatric use, the authors conclude that despite these animal studies, there have been no documented cases of fat embolism after IIC in infants and children [15]. Even in a more recent review about use of intraosseous needles in neonates, there is no mention of human studies [13].

To the best of our knowledge, our study is the first specifically aimed to verifying the occurrence of PFE after IIC by using autopsy data of a pediatric population. The results of our study seem to confirm that IIC can lead to PFE in a pediatric population and above all show that PFE after IIC can be important (up to score 2 of Falzi).

Concerning the pathophysiology of PFE, the studies on animal models suggest a mechanical origin, related to the fact that intraosseous infusion increases the intramedullary pressure and can lead to microscopic fractures of the metaphysis of the bone and vascular damage, with possible penetration of fat droplets in the blood circulation. This mechanism seems to be plausible to explain the presence of PFE in our pediatric cases, especially in relation to the smaller medullary cavity of children compared to adults.

According to the literature, PFE after CPR related to rib fractures is usually mild (score 1 of Falzi) and not considered a factor that can limit the effectiveness of the resuscitation [6, 7]. However, in our study, we detected an important PFE (score 2 of Falzi) in about 30% of children that received an intraosseous infusion. Although this score is usually not considered a potential cause of death in itself, it is questionable whether it can reduce the effectiveness of CPR, for example, by hindering the tissue oxygenation and/or increasing right ventricular postload [19]. In a 2001 study, Hasan et al. already concluded that clinical relevance of PFE after IIC was unclear and that more studies were necessary to describe this phenomenon more precisely [9].

## Conclusions

Our study suggests that IIC during CPR can lead to PFE in a pediatric population, possibly by an increase of the intramedullary pressure with microscopic bone fractures and penetration of fat droplets into the blood circulation.

Furthermore, about 30% of our cases that received IIC showed an important PFE with a score 2 of Falzi. This result requires questioning about the possible risk that PFE after IIC can reduce the effectiveness of the resuscitation in a pediatric population.

Considering the need to use multiple exclusion criteria, the fact that the number of pediatric forensic autopsies is low and that the cases without IIC are just a few, we were not able to review a consistent number of cases. However, considering the potential clinical implication of our results, we believe that further research is needed.
